# Efficacy and Safety of Fractional Laser Therapy for Androgenetic Alopecia: A Systematic Review and Meta‐Analysis

**DOI:** 10.1111/jocd.70909

**Published:** 2026-05-12

**Authors:** Cheng Chen, Shuangshuang Wang, Dezhao Bi, Ang Ching Wei, Shun Guo

**Affiliations:** ^1^ Affiliated Hospital of Nanjing University of Chinese Medicine Jiangsu Province Hospital of Chinese Medicine Nanjing China; ^2^ Nanjing University of Chinese Medicine Nanjing China; ^3^ Community Health Service Center Chengnan Street, Qingjiangpu District Huai'an China

**Keywords:** ablative fractionated lasers, AGA, androgenetic alopecia, laser, meta, non‐ablative fractionated lasers

## Abstract

**Objective:**

To systematically evaluate the efficacy and safety of fractional laser therapy in the treatment of androgenetic alopecia.

**Methods:**

A comprehensive literature search was conducted in PubMed, Web of Science, Embase, the Cochrane Library, and China National Knowledge Infrastructure (CNKI) from inception to November 2025. Eligible studies were selected according to the PICOS framework. The methodological quality of the included studies was assessed using the Cochrane Risk of Bias tool. Meta‐analysis was performed using R software.

**Results:**

A total of 15 studies involving 1097 patients were included. Meta‐analysis demonstrated that fractional laser therapy significantly improved hair density compared with control interventions (mean difference [MD] = 13.88, 95% confidence interval [CI]: 6.87–20.90, *p* < 0.001). Subgroup analysis revealed no significant difference in efficacy between ablative and non‐ablative fractional lasers (*p* = 0.7792), nor between different wavelengths (1565 nm, 1550 nm, 1927 nm; *p* = 0.7014). Combination therapy with laser and other treatments (e.g., minoxidil) showed a certain advantage over other treatments alone (MD = 9.74, *p* = 0.001). Adverse events were predominantly mild to moderate, including erythema, pain, and pruritus, all of which resolved spontaneously.

**Conclusion:**

Fractional laser therapy demonstrates marked therapeutic efficacy and a favorable safety profile in the treatment of androgenetic alopecia, making it a viable clinical treatment option. Future high‐quality studies are warranted to further validate its long‐term effectiveness and optimal treatment parameters.

## 1 Introduction

Androgenetic alopecia (AGA) is a common chronic, non‐scarring form of hair loss that affects both men and women worldwide. The condition is characterized by progressive miniaturization of hair follicles, manifested by a gradual shortening of the anagen phase, a continuous reduction in follicular size, and the transformation of thick, pigmented terminal hairs into thin, hypopigmented vellus hairs. Over time, advanced follicular atrophy ultimately leads to irreversible hair loss [1]. Epidemiological data indicate marked population differences in AGA prevalence, with White men being disproportionately affected, where approximately 30% develop clinical signs by the age of 30 years, increasing to 50% by 50 years and nearly 80% by 70 years [2].

Besides, AGA also exhibits pronounced sex‐related differences, with a substantially higher prevalence in men than in women. In recent years, the incidence of early‐onset AGA among young men has shown a concerning upward trend [3]. Beyond its dermatological manifestations, AGA has significant psychosocial consequences. Hair loss can negatively affect self‐image and is frequently associated with psychological distress, including anxiety and depressive symptoms, leading to impaired quality of life and social functioning [4].

At present, therapeutic options for AGA remain limited. The U.S. Food and Drug Administration (FDA) has approved only two pharmacological treatments, which are topical minoxidil and oral finasteride. Though both agents show suspension in disease progression and promote partial hair regrowth, their clinical efficacy often depends on long‐term continuous use, and treatment discontinuation frequently results in relapse. Likewise, these therapies may be accompanied by adverse effects, such as hypertrichosis and sexual dysfunction, which further limit patient adherence and acceptance in clinical practice [5].

Laser‐based therapies have increasingly attracted attention as alternative or adjunctive treatments in dermatology. Clinically available laser systems can be broadly categorized into four groups, including ablative non‐fractionated lasers, non‐ablative non‐fractionated lasers, non‐ablative fractional lasers, and ablative fractional lasers. Ablative lasers, including carbon dioxide (CO_2_, 10 600 nm) and erbium lasers (2940 nm), induce controlled tissue vaporization and are associated with longer recovery times and greater post‐treatment care requirements, but often yield more pronounced clinical effects. In contrast, non‐ablative lasers such as those operating at wavelengths of 1550, 1540, 1565, and 1927 nm preserve epidermal integrity, resulting in milder treatment responses with minimal downtime, albeit with relatively modest efficacy [6].

Fractional laser technology delivers laser energy in a pixelated pattern, creating microscopic thermal zones (MTZs) through selective photothermolysis. These controlled micro‐injuries stimulate tissue remodeling and regeneration while minimizing damage to surrounding structures, thereby offering improved safety and selectivity compared with conventional laser modalities [7].

In recent years, fractional lasers have been increasingly applied in the management of hair loss disorders [8]. However, existing clinical studies on fractional laser therapy for AGA have yielded heterogeneous results, and a comprehensive and systematic evaluation of its efficacy and safety remains lacking. Therefore, the present study aims to systematically evaluate the therapeutic efficacy and safety of fractional laser therapy for AGA through a systematic review and meta‐analysis, providing evidence‐based support for clinical decision‐making.

## 2 Methods

### 2.1 Literature Search

Two authors independently conducted a comprehensive literature search in PubMed, Web of Science, Embase, the Cochrane Library, and the China National Knowledge Infrastructure (CNKI) database. All databases were searched from their inception to November 2025. The search strategy combined Medical Subject Headings (MeSH) terms and free‐text keywords, including “androgenetic alopecia,” “alopecia,” “male pattern baldness,” “female pattern hair loss,” “fractional laser,” “laser,” “ablative fractional laser,” and “non‐ablative fractional laser.”

### 2.2 Inclusion and Exclusion Criteria

Eligibility criteria were defined according to the PICOS framework (Participants, Interventions, Comparisons, Outcomes, and Study design). Studies were included if they met the following criteria: (1) participants were adults aged 18 years or older with a clinical diagnosis of androgenetic alopecia; (2) the intervention involved fractional laser therapy used either as a monotherapy or as an adjunctive treatment for androgenetic alopecia, with at least one study arm receiving fractional laser treatment; (3) comparators included any type of control intervention or alternative treatment modality; (4) outcomes included measures of hair regrowth; and (5) eligible study designs comprised randomized controlled trials (RCTs), other controlled clinical trials, and cohort studies.

Studies were excluded if they met any of the following criteria: (1) studies employing non‐standard or methodologically flawed designs, or those judged to be of low quality; (2) studies primarily designed to compare different types of fractional lasers or other laser modalities; (3) studies with unspecified, incomplete, or unavailable outcome data; and (4) duplicate publications.

### 2.3 Data Extraction

Two reviewers independently screened the titles, abstracts, and keywords of all retrieved records to identify potentially eligible studies. Full texts of relevant articles were then assessed in detail to determine final eligibility according to the predefined inclusion criteria. Any disagreements between the two reviewers were resolved through discussion with a third reviewer.

Data extracted from each included study comprised the first author, year of publication, study design, sample size, participant characteristics, details of interventions in the experimental and control groups, specific parameters of fractional laser therapy including laser type, wavelength, energy density, number of treatment sessions, and treatment intervals, outcome measures, and reported adverse events.

### 2.4 Risk‐of‐Bias Assessment

The methodological quality of the included studies was assessed using the Cochrane Risk of Bias tool (RoB 2.0). Several domains were evaluated including bias arising from the randomization process, deviations from intended interventions, missing outcome data, measurement of outcomes, and selection of the reported results. Each domain was judged as having a “low risk,” “high risk,” or “some concerns” of bias.

### 2.5 Outcome Measurement

Given the heterogeneity in clinical outcome assessment methods across the included studies, hair density measured by dermoscopic evaluation before and after treatment was selected as the primary outcome measure.

### 2.6 Statistical Analysis

Statistical analyses were performed using the meta package in R software (version 4.4.3). Between‐study heterogeneity was assessed using the *I* ^2^ statistic. Significant heterogeneity was considered present when *I* ^2^ exceeded 50% or when the *p* value was < 0.10, in which case a random‐effects model was applied; otherwise, a fixed‐effects model was used.

Subgroup analyses were conducted based on sources of clinical heterogeneity, including laser type, wavelength, and type of comparison. Publication bias was evaluated using funnel plots and Egger's regression test. Sensitivity analysis was performed by sequentially excluding each individual study to assess the robustness of the pooled results. A *p* value of < 0.05 was considered statistically significant.

## 3 Results

### 3.1 Literature Search and Study Characteristics

**FIGURE 1:**
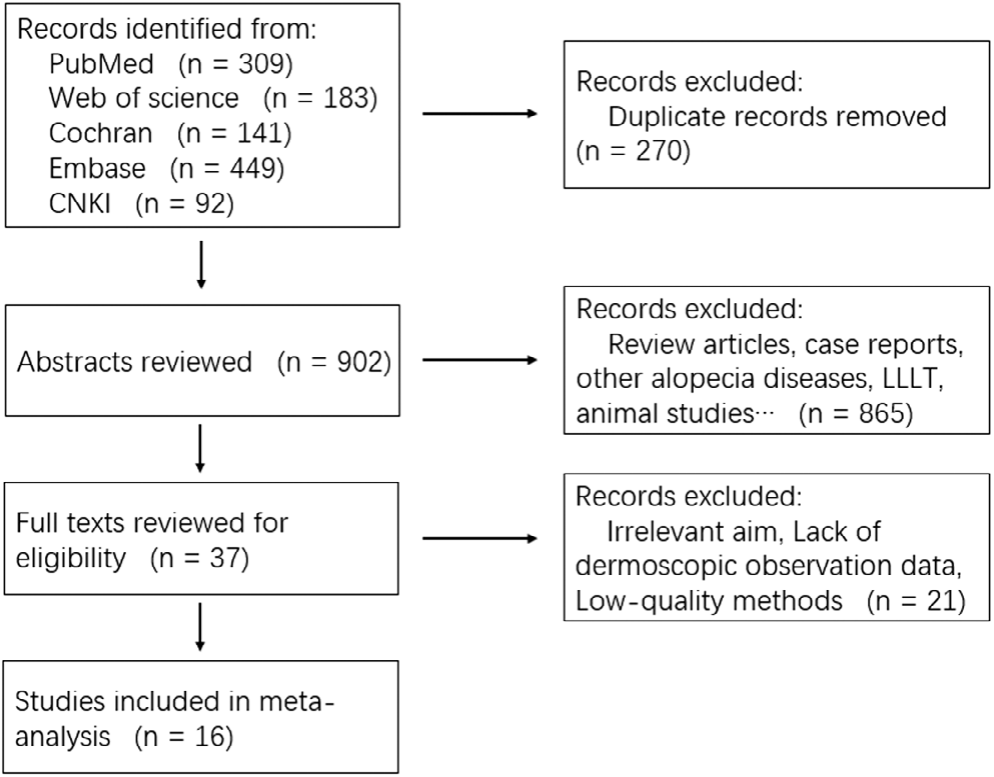
Flowchart of study search and inclusion criteria.

The initial literature search identified a total of 1172 records from PubMed, Web of Science, Embase, the Cochrane Library, and CNKI. After a stepwise screening process, 15 studies involving 1097 patients were ultimately included in the analysis. The study selection process is illustrated in Figure 1, and the main characteristics of the included studies are summarized in Table 1.

**TABLE 1:** Basic characteristics of the included studies.

Author, year	Study design	No. subjects (male/female)	Laser type	Laser settings	Number of treatment sessions, treatment interval	Group	Adverse effects	Outcome measurements
Chen, 2025 [9]	RCT	75 (75/0)	CO_2_	Pulse energy—10 mJ Coverage—6.3% Spacing—0.3 ~ 0.6 mm	Sessions—12 Interval—1 week	1. CO_2_ + minoxidil 2. Microneedles + minoxidil 3. Minoxidil	Pain, Itching, Erythema	1. Dermoscopy 2. Subjective Symptom Score 3. Clinical Efficacy Assessment (Weiss Criteria)
Cheng, 2024 [10]	RCT	75 (75/0)	Non‐ablative fractional laser (1565 nm)	Pulse energy—30 ~ 40 mJ Density—200 spots/cm^2^	Sessions—6 Interval—4 weeks	1. minoxidil 2. minoxidil + finasteride 3. minoxidil + finasteride + 1565 nm	Pain, Itching, Erythema, Decreased libido	1. Dermoscopy 2. Subjective Symptom Score 3. Clinical Efficacy Assessment
Li, 2025 [11]	Randomized, single‐blind, self‐controlled clinical trial	62 (62/0)	Er:YAG laser (2940 nm)	Mode: Long pulse width Energy: 600 mJ/cm^2^ Spot size: 9 × 9 mm	Sessions—12 Interval—2 weeks	1. minoxidil 2. minoxidil + 2940 nm	Pain, Erythema, Itching, Increased dandruff	1. Dermoscopy 2. Clinical Grading 3. Expert Assessment 4. Patient Satisfaction
Li, 2024 [12]	RCT	114 (73/41)	CO_2_	Pulse energy—30 ~ 40 mJ/cm^2^	Sessions—8 Interval—3 weeks	1. minoxidil 2. minoxidil + CO_2_	Scalp redness, Bleeding, Ecchymosis, Dermatitis papules	1. Dermoscopy 2. Clinical symptom improvement score 3. Treatment efficacy scores 4. Quality of life assessments
Yang, 2025 [13]	RCT	80 (80/0)	Non‐ablative fractional laser (1565 nm)	Pulse energy: 10–20 mJ Coagulation depth: 327–417 μm Coagulation width: 87–103 μm	Sessions—10 Interval—2 weeks	1. minoxidil + finasteride 2. minoxidil + finasteride + 1565 nm	Increased dandruff, Scalp pruritus, Scalp burning, Local rash	1. Dermoscopy 2. Clinical efficacy
Tan, 2024 [14]	RCT	90 (0/90)	Erbium laser (2940 nm)	Matrix size: 13 × 13 spots Pulse rate: 4 pulses per second Energy density: 50–75 J/cm^2^	Sessions—3 Interval—1 month	1. PRP* + 2940 nm 2. minoxidil + 2940 nm 3. 2940 nm	Increased greasiness, Increased dandruff, Itching, Erythema	1. Dermoscopy 2. Clinical Efficacy 3. Subjective Symptom Scores 4. Patient Satisfaction
Hu, 2022 [15]	RCT	30 (18/12)	Erbium laser (2940 nm)	Spot size: 7 mm × 7 mm Energy: 800 mJ/cm^2^ Mode: Long pulse width	Sessions—6 Interval—2 weeks	1. Minoxidil + 2940 nm 2. PRP 3. Minoxidil + 2940 nm + PRP	Scalp pruritus	1. Dermoscopy 2. Clinical Efficacy 3. Subjective Symptom Scores
Wen, 2024 [16]	RCT	102 (gender distribution not specified in the article)	Erbium laser (2940 nm)	Spot size: 7 mm × 7 mm Energy: 600 mJ/cm^2^ Mode: Long pulse width	Sessions—3 Interval—1 month	1. 2940 nm 2. 2940 nm + PRP 3. 2940 nm + i‐PRF*	None	1. Dermoscopy 2. Clinical Efficacy 3. Subjective Symptom Scores
Tan, 2023 [17]	Clinical trial	80 (80/0)	Erbium laser (2940 nm)	Spot size: 7 mm × 7 mm Energy: 200 mJ/cm^2^ Mode: Long pulse width	Sessions—6 Interval—2 weeks	1. Minoxidil + finasteride 2. Minoxidil + finasteride + 2940 nm 3. Minoxidil + finasteride + PRP	None	1. Dermoscopy 2. Clinical Efficacy 3. Subjective Symptom Scores
Suchonwanit, 2019 [18]	Randomized, investigator‐blinded, controlled, split‐scalp study	30 (30/0)	Er:Glass laser (1550 nm)	Energy: 6 mJ Density: 300 spot/cm^2^	Sessions – 12 Interval—2 weeks	1. Minoxidil + 1550 nm 2. Minoxidil	Erythema, Itching, Scaling	1. Dermoscopy 2. Global photographic assessment
Ma, 2023 [19]	RCT	60 (60/0)	CO_2_	Energy: 10–15 mJ/cm^2^ Spot density: 10%	Sessions – 6 Interval—1 month	1. Minoxidil 2. Minoxidil + finasteride 3. Minoxidil + finasteride + CO_2_	Erythema, Scaling, Decreased libido, Pruritus	1. Dermoscopy 2. Clinical Efficacy
Dai, 2024 [20]	Retrospective, single‐center cohort study	192 (192/0)	Er:YAG laser (2940 nm)	Energy: 20–30 J/cm^2^ Pulse time: 300 μs	Sessions – 6 Interval—2 weeks	1. Minoxidil + finasteride + 2940 nm 2. Minoxidil + finasteride	Erythema, Pruritus	1. Dermoscopy 2. Global photographic assessment
Huang, 2017 [21]	Randomized half‐split study	27 (27/0)	CO_2_	Energy: 12–18 mJ/spot Spot density: 361 spots/cm^2^	Sessions – 6 Interval—2 weeks	1. CO_2_ + bilateral hair growth factors 2. Bilateral hair growth factors	Erythema, Slight edema, Pruritus, Dryness, Seborrheic dermatitis, Dandruff	1. Dermoscopy 2. Global photographic assessment 3. Global Photography Assessment
Qu, 2024 [22]	Investigator‐blinded, controlled, randomized comparative study	30 (14/16)	Non‐ablative fractional laser (1565 nm)	Energy: 10 mJ Spot density: 250 spots/cm^2^ Overlap: 3% Passes: 1 path	Sessions – 4 Interval—2 weeks	1. 1565 nm 2. Minoxidil	Pain, Itching	1. Dermoscopy 2. Patient satisfaction score 3. Global photographic assessment
Cho, 2018 [23]	Split‐scalp unblinded RCT	10 (10/0)	Thulium laser (1927 nm)	Energy — 4 mJ/6 mJ Spot size — 100 μm Spot density—100–140 spot/cm^2^	Sessions – 12 Interval—1 weeks	1. Thulium + growth factors 2. Thulium	Transient erythema, Pruritus	1. Dermoscopy 2. Patient satisfaction score

Abbreviations: i‐PRF, injectable platelet‐rich fibrin; PRP, Platelet‐rich plasma.

### 3.2 Quality Assessment

The methodological quality of the included studies was evaluated using the Cochrane Risk of Bias tool. With respect to the randomization process, two studies were judged to be at high risk of bias, while thirteen studies were assessed as having a low risk. For the remaining domains including deviations from intended interventions, missing outcome data, measurement of outcomes, and selection of the reported results, all fifteen studies were rated as having a low risk of bias.

**FIGURE 2:**
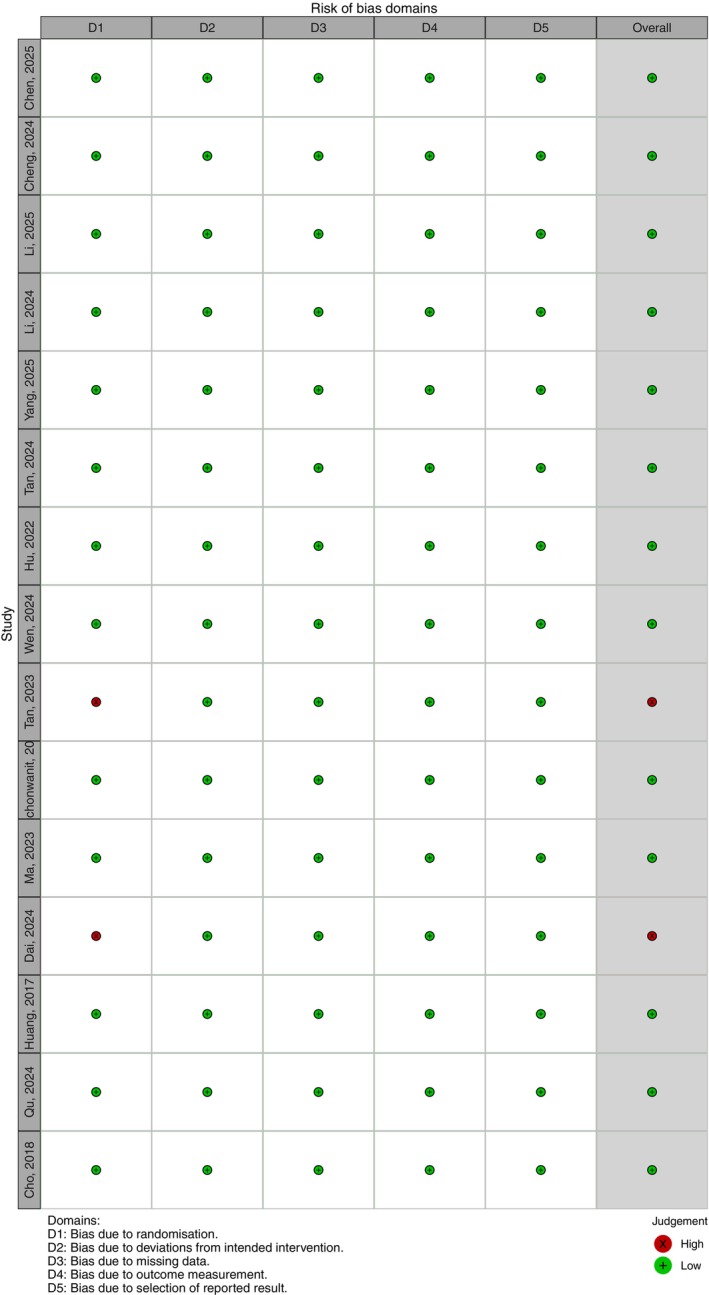
Risk of bias of included randomized controlled trials (green: Low risk; red: High risk).

Overall, two studies were classified as having a high risk of bias and thirteen as having a low risk of bias. Taken together, the overall quality of the included studies was considered moderate. A detailed summary of the risk of bias assessment is presented in Figure 2.

### 3.3 Quantitative Meta‐Analysis

#### 3.3.1 Overall Meta‐Analysis

**FIGURE 3:**
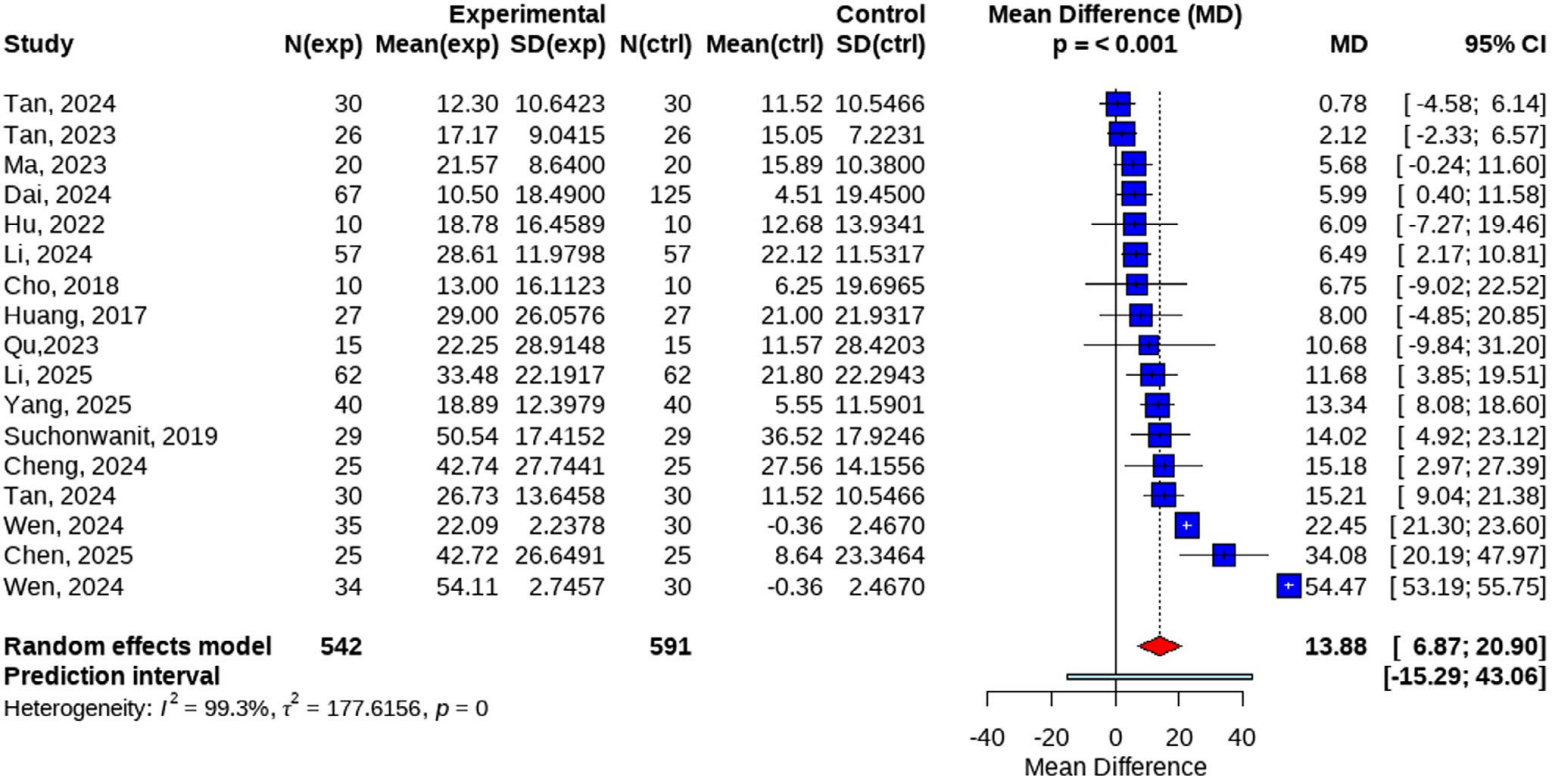
Forest plot of efficiency rate in overall.

A total of 15 studies were included in this meta‐analysis. Among them, two studies contributed multiple independent datasets, resulting in a total of 17 datasets being pooled for the overall analysis. Using a random‐effects model, the meta‐analysis demonstrated a significant improvement in the primary outcome in the treatment group compared with the control group, with a mean difference (MD) of 13.88 (95% confidence interval [CI]: 6.87–20.90, *p* < 0.001). Substantial heterogeneity was observed among the included studies (*I* ^2^ = 99.3%, *p* < 0.001) (Figure 3).

#### 3.3.2 Subgroup Analysis

When subgroup analyses were performed according to laser type, 12 datasets were included in the ablative fractional laser subgroup. The pooled analysis demonstrated a significant improvement in the primary outcome for the treatment group compared with the control group, with a mean difference (MD) of 14.46 (95% CI: 4.34–24.57, *p* = 0.009). However, substantial heterogeneity was observed within this subgroup (*I* ^2^ = 99.5%).

**FIGURE 4:**
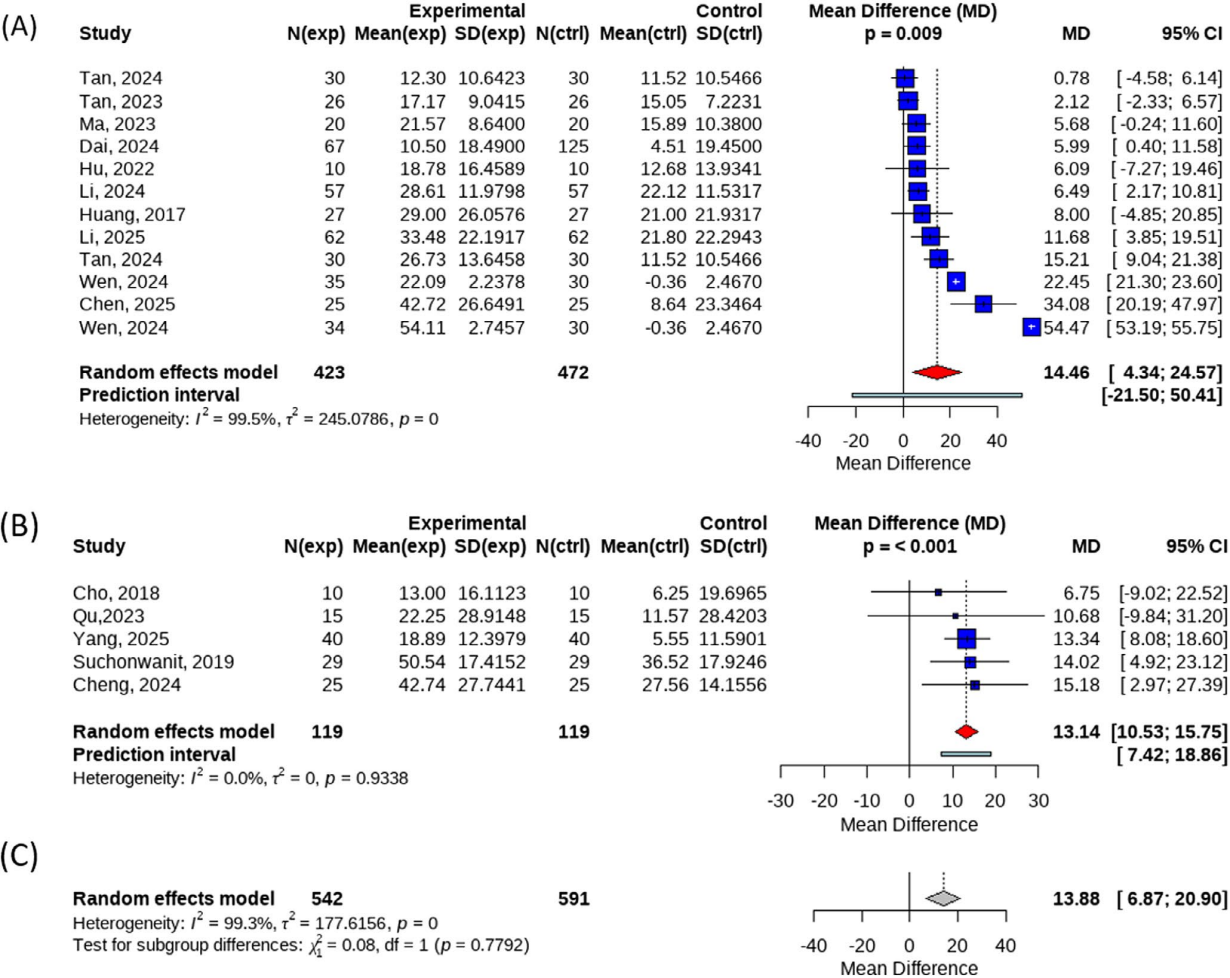
(A) Forest plot of efficiency rate in subgroup of Ablative Fractionated Lasers; (B) Forest plot of efficiency rate in subgroup of Non‐ablative Fractionated Lasers; (C) Forest plot for the between‐group comparison between ablative and non‐ablative fractional laser groups.

Five datasets were included in the non‐ablative fractional laser subgroup. The results showed a significant benefit of treatment over control, with an MD of 13.14 (95% CI: 10.53–15.75, *p* < 0.001), and no heterogeneity was detected (*I* ^2^ = 0%). The between‐subgroup comparison revealed no statistically significant difference in efficacy between ablative and non‐ablative fractional lasers (*p* = 0.7792) (Figure 4).

**FIGURE 5:**
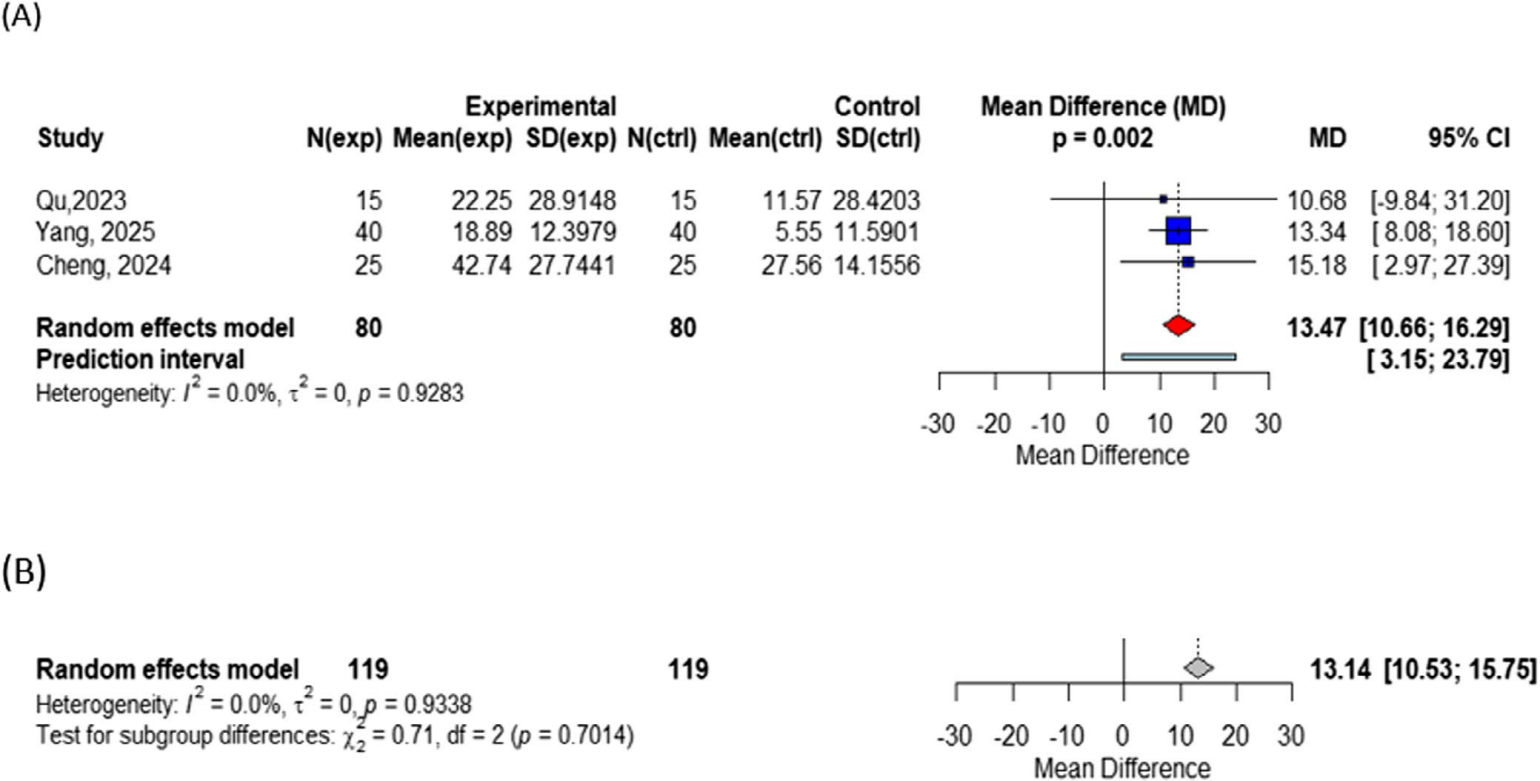
(A) Forest plot of efficiency rate in subgroup of 1565 nm wavelength; (B) Forest plot for the between‐group comparison among different wavelengths (1565 nm, 1550 nm, and 1927 nm).

To further investigate the differences in efficacy among various wavelengths of non‐ablative fractional lasers, we conducted an additional analysis. Within the non‐ablative fractional laser subgroup, three wavelengths were identified: 1565 nm, 1550 nm, and 1927 nm. Three datasets were included in the efficacy analysis for the 1565 nm wavelength, and the pooled results showed a mean difference in the primary outcome of 13.47 (95% CI: 10.66–16.29) between the treatment and control groups, with a statistically significant difference (*p* = 0.002) and no heterogeneity detected (*I* ^2^ = 0%). For the 1550 nm and 1927 nm wavelengths, only one dataset each was included in the analysis, with effect sizes of 14.02 (95% CI: 4.92–23.12) and 6.75 (95% CI: −9.02 to 22.52), respectively. Due to the limited number of studies, quantitative pooling was not performed for these two wavelengths. The test for between‐group differences yielded a *p* value of 0.7014, indicating that the differences in efficacy among the three wavelength groups were not statistically significant (Figure 5).

Subgroup analyses were further conducted according to treatment modality. Four datasets compared fractional laser therapy combined with other treatments versus fractional laser therapy alone. The pooled analysis showed an MD of 23.34 (95% CI: −12.82 to 59.50), which did not reach statistical significance (*p* = 0.132) and exhibited substantial heterogeneity (*I* ^2^ = 99.8%).

**FIGURE 6:**
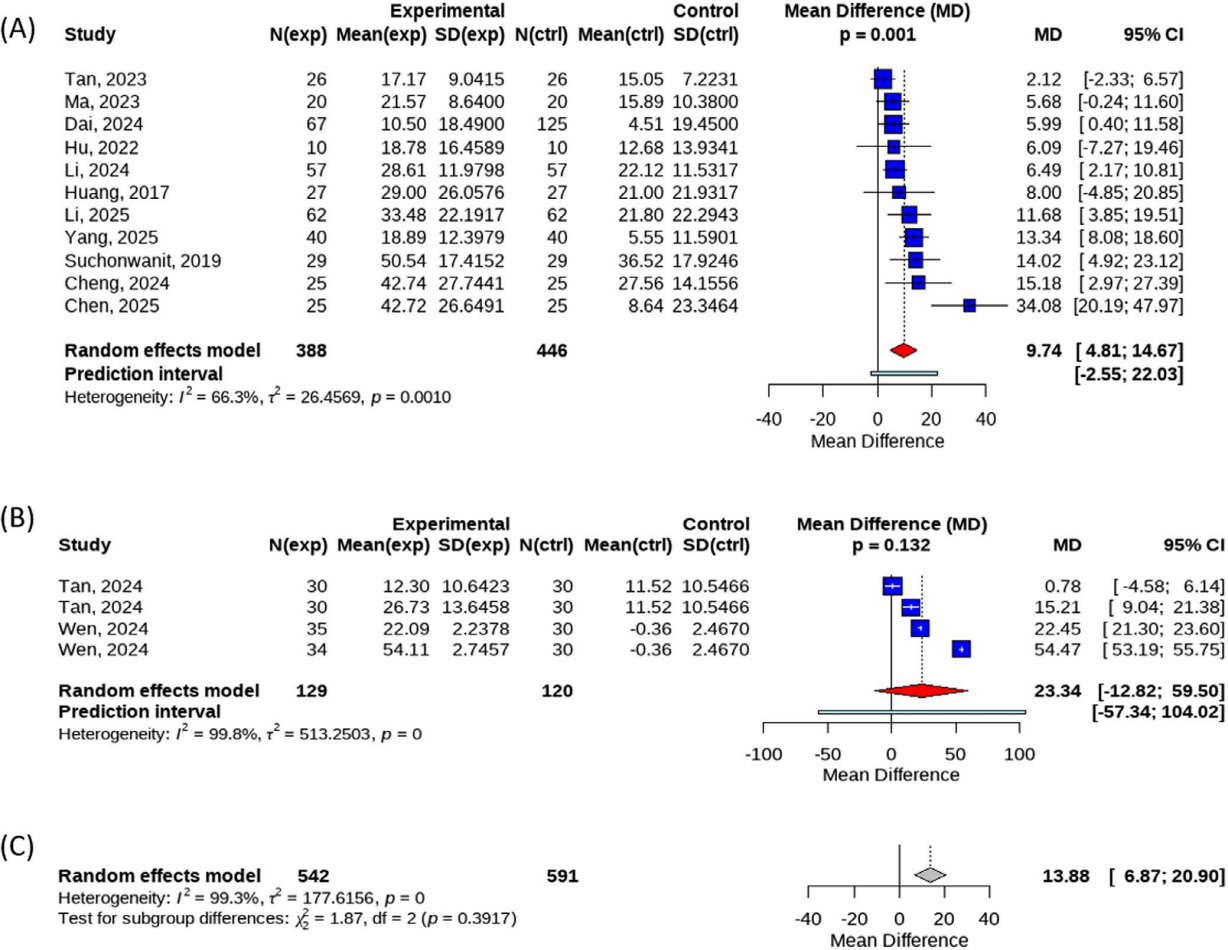
(A) Forest plot of efficiency rate in subgroup of Laser + Other therapy VS Other therapy; (B) Forest plot of efficiency rate in subgroup of Laser + Other therapy vs Laser; (C) Forest plot for the between‐group comparisons of the three treatments.

Eleven datasets compared fractional laser therapy combined with other treatments versus other treatments alone. This analysis demonstrated a statistically significant improvement in the primary outcome for the combination therapy group, with an MD of 9.74 (95% CI: 4.81–14.67, *p* = 0.001), accompanied by moderate heterogeneity (*I* ^2^ = 66.3%). Only two datasets compared fractional laser therapy alone with other treatments alone, yielding effect sizes of 6.75 (95% CI: −9.02 to 22.52) and 10.68 (95% CI: −9.84 to 31.20), respectively. Owing to the limited number of studies, quantitative pooling was not performed. The between‐subgroup comparison showed no statistically significant difference among the three treatment modalities (*p* = 0.3917) (Figure 6).

### 3.4 Assessment of Publication Bias

**FIGURE 7:**
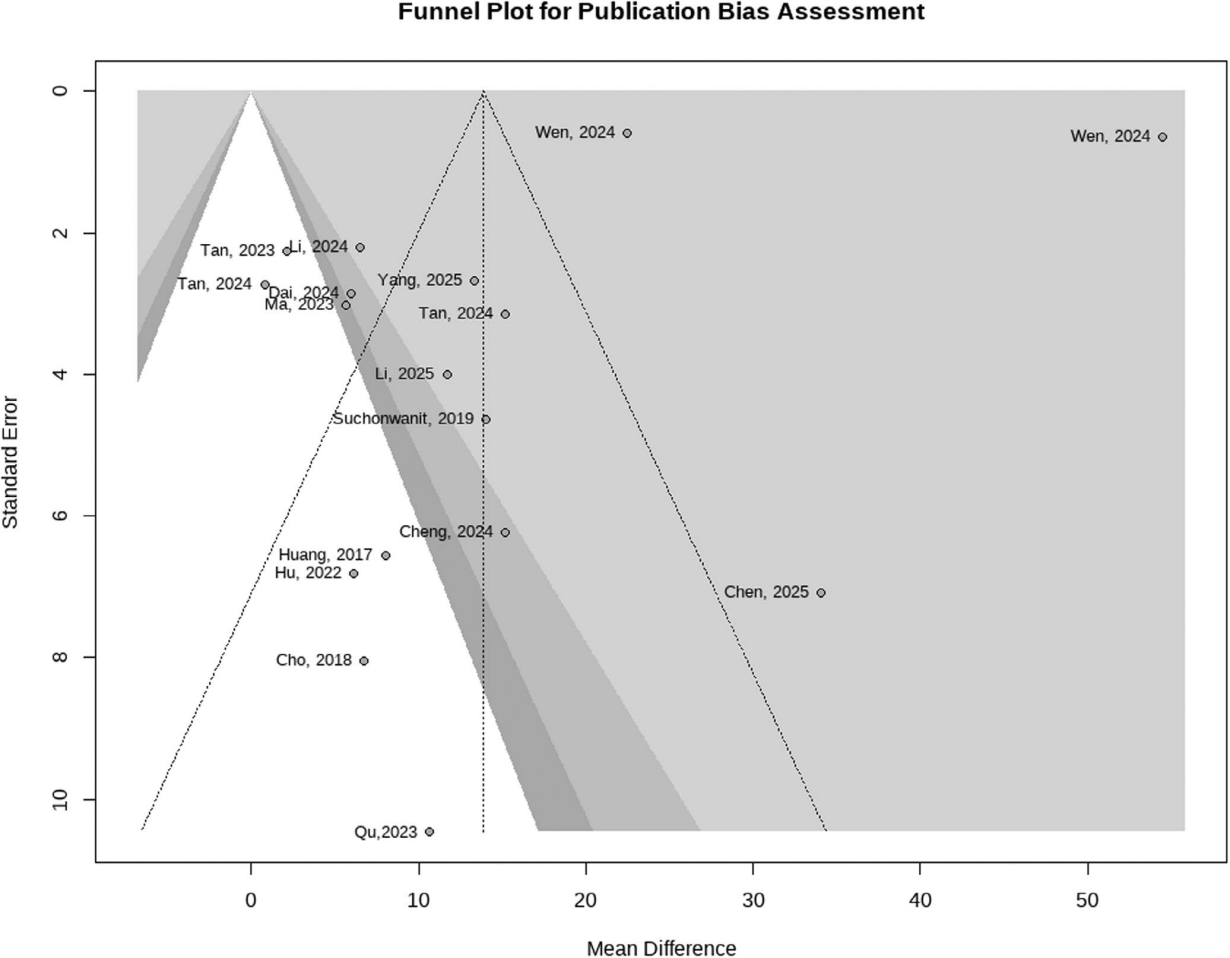
Funnel plot for publication bias assessment.

To further assess publication bias, Egger's linear regression test was performed. The results yielded an intercept of −7.37 (95% CI: −14.94 to 0.19), with a *p* value of 0.0553. Although this value did not reach the conventional threshold for statistical significance (*p* < 0.05), it was below the commonly used screening threshold for potential publication bias (*p* < 0.10), suggesting the possibility of mild publication bias. Consistent with this finding, visual inspection of the funnel plot (Figure 7) revealed a degree of asymmetry, indicating that the results of the present study may be influenced by some degree of publication bias, although this did not reach conventional statistical significance.

### 3.5 Sensitivity Analysis

**FIGURE 8:**
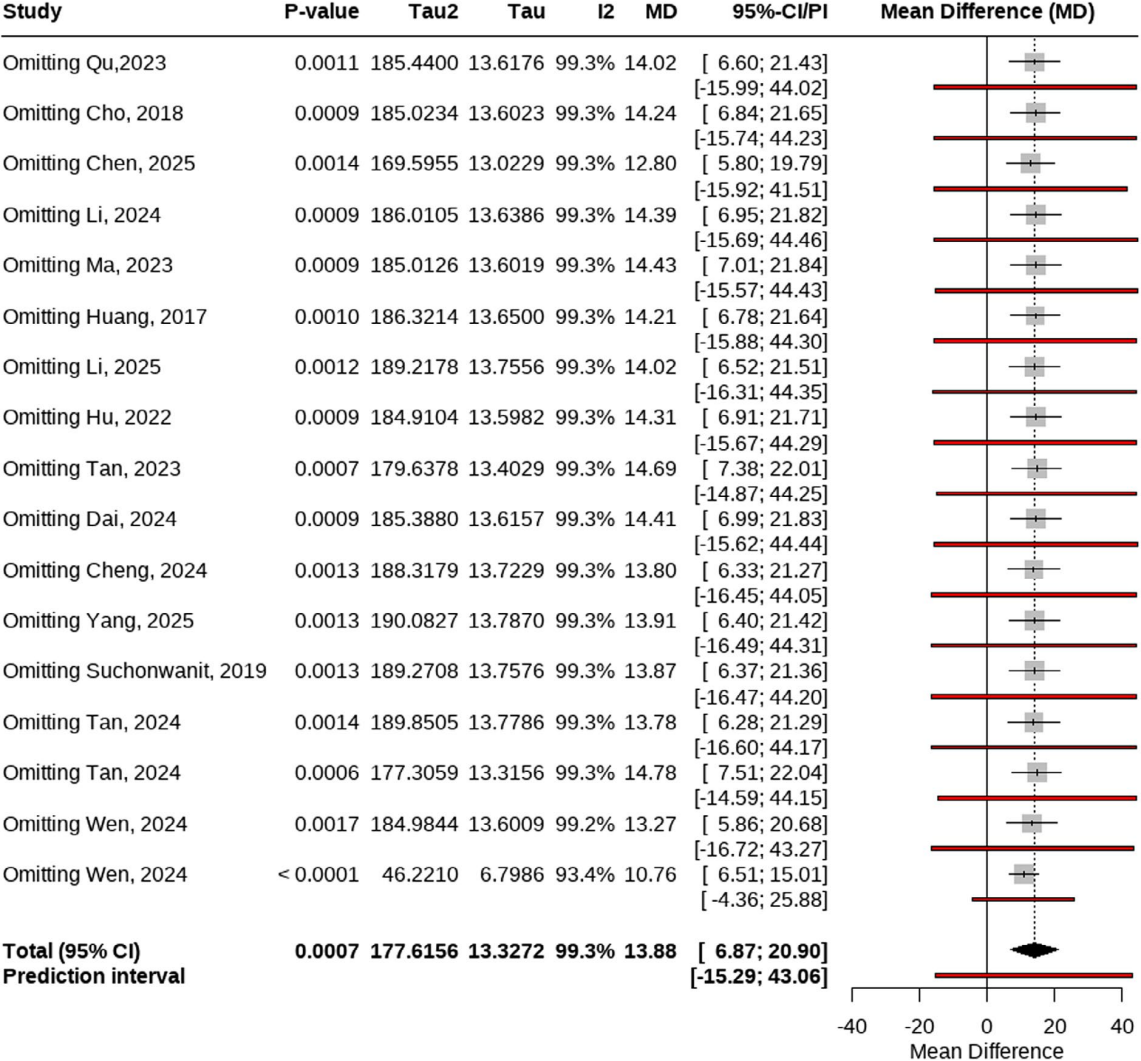
Forest plot for sensitivity analysis.

To assess the robustness of the meta‐analysis results, a sensitivity analysis was performed by sequentially excluding each individual study and recalculating the pooled effect estimates. The sensitivity analysis forest plot (Figure 8) showed that the overall effect size remained largely unchanged after the exclusion of any single study, and all corresponding *p* values remained statistically significant (*p* < 0.05), further confirming the stability of the results.

### 3.6 Side Effects

Adverse events reported in the included studies were mainly limited to erythema, pain, and pruritus. All adverse events were mild to moderate in severity and resolved spontaneously within 1–2 weeks without the need for specific treatment. No serious adverse events were reported.

## 4 Discussion

This meta‐analysis included 15 studies involving a total of 1097 patients with AGA. The pooled results demonstrated that fractional laser therapy was significantly more effective than control interventions in improving hair density (MD = 13.88, *p* < 0.001), indicating a favorable clinical effect of fractional laser treatment for AGA. These findings are generally consistent with those of previous studies and provide evidence‐based support for the use of fractional laser therapy as a treatment option for AGA.

Subgroup analyses based on laser type revealed no statistically significant difference in efficacy between ablative and non‐ablative fractional lasers (*p* = 0.7792), suggesting that both modalities may confer comparable benefits in terms of hair density improvement. However, substantial heterogeneity was observed within the ablative fractional laser subgroup (*I* ^2^ = 99.5%), which may be attributable to variations in laser parameters such as energy settings and treatment frequency, differences in treatment protocols, and heterogeneity in baseline patient characteristics. In contrast, the non‐ablative fractional laser subgroup exhibited no detectable heterogeneity (*I* ^2^ = 0%), indicating greater consistency across studies and potentially more stable treatment effects.

In terms of wavelength, no statistically significant difference in efficacy was observed among the different wavelength groups (1565 nm, 1550 nm, and 1927 nm; *p* = 0.7014). Notably, the 1565 nm subgroup demonstrated high within‐group consistency (*I* ^2^ = 0%), suggesting that 1565 nm may represent a relatively stable and reliable wavelength option among non‐ablative fractional lasers. However, given that both the 1550 nm and 1927 nm subgroups each included only a single study, the available data were insufficient to draw definitive conclusions regarding these wavelengths.

With respect to treatment strategies, fractional laser therapy combined with other treatments, such as minoxidil, demonstrated a statistically significant advantage over other treatments alone (MD = 9.74, *p* = 0.001), suggesting a potential synergistic effect of combination therapy. Nevertheless, the number of included studies was limited, particularly for direct comparisons between fractional laser monotherapy and other monotherapies. Therefore, the relative efficacy of different treatment strategies cannot yet be definitively established, and further well‐designed studies with larger sample sizes are warranted to identify optimal treatment regimens.

On the other hand, several limitations of this study should be acknowledged. Considerable heterogeneity was present among the included studies, and the results of Egger's test suggested the possibility of mild publication bias (*p* = 0.0553). In addition, substantial variability in laser parameters such as wavelength, energy settings, number of treatment sessions, and treatment intervals may have affected the comparability and consistency of the pooled outcomes. Despite these limitations, sensitivity analyses demonstrated that the overall effect estimates remained robust following sequential exclusion of individual studies, supporting the stability and reliability of the main findings.

In terms of operational procedures and precautions, prior to treatment, all fractional laser procedures require the treatment area to be kept clean and dry, avoiding areas with ulceration or inflammation. For ablative fractional laser treatment, topical lidocaine may be applied to alleviate intraoperative pain. Regarding energy selection, the energy dose should be adjusted according to the immediate tissue response observed during treatment to minimize adverse reactions and patient discomfort. Additionally, the laser coverage density should be controlled to prevent excessive local energy delivery. During the procedure, treatment should be confined to the hair loss area, with the hair parted along the follicular rows to avoid compressing hair against the scalp, which could lead to hair burning or breakage. Post‐treatment, local ice application may be used to alleviate burning and pain sensations, and patients should be advised to avoid sun exposure, friction, and contact with irritating substances.

In terms of adverse events, fractional laser therapy is primarily associated with mild to moderate erythema, pain, and pruritus. Following ablative fractional laser treatment, localized swelling, exudation, and crusting may occur; however, these reactions typically resolve spontaneously within a short period. No serious adverse events were reported, indicating a favorable safety profile for its clinical application.

## 5 Conclusion

This systematic review and meta‐analysis demonstrate that fractional laser therapy is an effective and safe treatment option for androgenetic alopecia, with adverse events that are generally mild and transient. The therapeutic efficacy of ablative and non‐ablative fractional lasers appears to be comparable, and similar efficacy was observed among different wavelengths (1565 nm, 1550 nm, and 1927 nm). However, combination therapy with other treatment modalities may further enhance clinical outcomes. Future research should focus on conducting high‐quality, large‐scale randomized controlled trials with long‐term follow‐up to clarify optimal treatment parameters, assess long‐term efficacy, and better define the role of fractional laser therapy within comprehensive management strategies for androgenetic alopecia.

## Author Contributions

C.C. and S.W.: article selection, statistical analysis, and manuscript drafting. D.B.: data extraction, statistical analysis. A.C.W.: plot. S.G.: supervision.

## Funding

This work was supported by the National Natural Science Foundation of China (Project No. 81603626). Jiangsu Pharmaceutical Association—Jin Peiying Foundation Project (J2024020). The funder had no role in the design, data collection, data analysis, and reporting of this study.

## Conflicts of Interest

The authors declare no conflicts of interest.

## Data Availability

The data that supports the findings of this study are available in the Supporting Information of this article.
